# Gestational diabetes mellitus and cardio-metabolic risk factors in women and children at 3 years postpartum

**DOI:** 10.1007/s00592-022-01914-y

**Published:** 2022-07-11

**Authors:** Maleesa M. Pathirana, Prabha H. Andraweera, Emily Aldridge, Shalem Y. Leemaqz, Madeline Harrison, Jade Harrison, Petra E. Verburg, Margaret A. Arstall, Gustaaf A. Dekker, Claire T. Roberts

**Affiliations:** 1 Level 6, Adelaide Health and Medical Sciences Building, The University of Adelaide, Adelaide, SA 5005 Australia; 2 Robinson Research Institute, The University of Adelaide, Adelaide, SA 5005 Australia; 3 Department of Cardiology, Lyell McEwin Hospital, Haydown Road, Elizabeth Vale, SA 5112 Australia; 4 Flinders Health and Medical Research Institute, Flinders University, Bedford Park, SA 5042 Australia; 5 Department of Obstetrics and Gynaecology, Lyell McEwin Hospital, Haydown Road, Elizabeth Vale, SA 5112 Australia

**Keywords:** Gestational diabetes, Child health, Cardiovascular disease, Maternal health

## Abstract

**Introduction:**

Gestational diabetes mellitus (GDM) is thought to be associated with cardio-metabolic risk factor development in women and their children during the early postpartum period and early childhood. We hypothesized that these women and their children would exhibit increased abnormal cardio-metabolic risk factors three years after pregnancy.

**Methods:**

Women from the Screening Tests to Predict Poor Outcomes of Pregnancy study were invited to attend a follow-up with the child from their index pregnancy at 3 years postpartum. Women and children were assessed for anthropometric measures and haemodynamic function. Fasting blood samples were obtained from women to assess lipid and glucose status.

**Results:**

A total of 281 woman-child dyads participated in the 3-year follow-up, with 40 women developing GDM during their index pregnancy. Fasting serum insulin was higher in women with GDM in index pregnancy compared to those with an uncomplicated pregnancy. However, this association was mediated by early pregnancy BMI and socioeconomic index (SEI). The rate of metabolic syndrome was higher in the GDM group than the uncomplicated pregnancy group. Maternal GDM was associated with elevated maternal fasting serum triglycerides at 3 years after adjustment for early pregnancy BMI and SEI. Children exposed to GDM in utero had higher waist circumference compared to children born after an uncomplicated pregnancy, but this is mediated the above covariates.

**Conclusion:**

Exposure to GDM is associated with elevated serum triglycerides in women at 3 years postpartum but other cardiometabolic outcomes in women and children appear to be mediated by early pregnancy BMI and SEI.

**Supplementary Information:**

The online version contains supplementary material available at 10.1007/s00592-022-01914-y.

## Introduction

Cardiovascular disease (CVD) is the number one cause of global mortality, with 17.9 million deaths in 2016, representing 31% of all global deaths in that year [1]. The Australian Institute of Health and Welfare reported that 78% of CVD burden for females in 2015 was considered ‘fatal’ death due to premature death [2]. Therefore, it is important to understand causes and risk factors for CVD that put women at an increased risk.

Gestational diabetes mellitus (GDM) is defined as de novo diagnosis of diabetes during pregnancy [3]. It is commonly diagnosed at 24–28 weeks’ gestation but prior risk factors including family history can qualify a woman to be tested earlier [4]. Having GDM increases risk of developing type 2 diabetes mellitus (T2DM) by 50% within five years post pregnancy, placing young women at increased risk of premature coronary heart disease [5]. Understanding the absolute cardiovascular risk for this group of women allows for early intervention and merits further research. Elevated blood pressure, serum triglycerides, blood glucose, which together are part of the diagnostic criteria for metabolic syndrome, have been detected within the first 12 months postpartum in women with a history of GDM [6]. Metabolic syndrome is a CVD risk factor and seen in women and children exposed to GDM [7].

Offspring who are exposed to GDM in utero exhibit higher systolic blood pressure than their counterparts who were not exposed [8]*.* Staley et al*.* demonstrated blood pressure differences between offspring of women who developed hypertensive disorders of pregnancy compared to those from normotensive mothers consistently throughout childhood and adolescence [9]. Therefore, offspring exposed to GDM in utero may exhibit anthropometric and/or cardiovascular changes at an earlier age.

Our primary aim was to assess cardiovascular risk factors in women with and normoglycemic recruited from a socioeconomically disadvantaged community. Our secondary aim was to assess these risk factors in their children at age 3. As an exploratory aim, we assessed the effect of maternal early pregnancy obesity on these cardiovascular risk factors in both women with a history of GDM and their children at 3 years postpartum.

## Methods

### Study population

The study participants included women and their children from the Screening Tests to Predict Poor Outcomes of Pregnancy (STOP) study recruited in pregnancy in 2015 to 2017 [10]. The STOP study was a prospective cohort study that aimed to assess and predict the risk for pregnancy complications. A total of 1,363 nulliparous women, their partners and babies were originally recruited. Majority of the participants were recruited from the Northern Adelaide Local Health Network which serves a community residing in one of the most socioeconomically disadvantaged regions in metropolitan Australia [11]. This community harbours some of the highest rates of diabetes, heart disease and mental illness. Women of the STOP follow-up study were contacted using phone numbers provided during the STOP study, or from hospital records. Ethics approval was granted by the Central Adelaide Local Health Network (STOP study: (HREC/14/WCHN/90) (ACTRN12614000985684), STOP follow-up study: HREC 18/CAHLN/318).

## Clinical data

STOP study included data of only nulliparous women collected at 9–16 and 32–36 (mean 34) weeks’ gestation and following delivery of the baby. Data on demography, medical and family history were collected. Socioeconomic index (SEI) was assessed using the New Zealand Socioeconomic Index (NZSEI) [12]. Physical measurements including height, weight, waist circumference, BMI and haemodynamic measurements were performed. GDM was diagnosed at 24–28 weeks’ gestation according to the International Association of Diabetes in Pregnancy Study Group (IADPSG) criteria [i.e. one or more values equal to or exceeding: fasting plasma glucose of 5.1 mmol/L, and/or a 2 h plasma glucose level of 8.5 mmol/l following a 75 g Oral Glucose Tolerance Test (OGTT)] [13]. Women who were at high risk of GDM also completed a 75 g OGTT in their first trimester. Data collected at birth included newborn weight, length, arm circumference, birthweight centile, and data on complications during the neonatal period.

Women were recruited into the STOP follow-up study within 3 months (either side) of when their first child turned 3 years old. Women residing regionally or interstate consent remotely to participating in the follow-up study, and complete anthropometric, haemodynamic and serum biochemistry through their general practitioner. Appointments were completed at the Clinical Trials Unit at the Lyell McEwin Hospital. Height of women and children was measured with a stadiometer to the nearest 0.1 cm. Children’s weight was measured with a standard balance beam scale to the nearest 100 g. Body composition in women was assessed using the TANITA SC-330 bioimpedance scale (Tokyo, Japan) which measured fat mass to the nearest 0.1 kg, fat percentage, fat mass, fat free mass and BMI. Body composition in children was assessed by standardized BMI score based on the Centre for Disease Control (CDC) growth charts for children and teenagers aged 2 to 19 years of age [14]. Waist circumference was measured in both women and children to the nearest 0.5 cm [15]. Peripheral systolic, diastolic and mean arterial blood pressure was assessed using the USCOM BP + (USCOM, Sydney, Australia) using appropriately sized cuffs for arm circumference, while participants were rested for at least 20 min and seated. The USCOM BP + was used to perform several non-invasive measures of cardiovascular function, including pulse rate, peripheral systolic and diastolic blood pressures, central systolic and diastolic blood pressures, which reflect blood pressure in the aorta and functionality of the heart, and augmentation index (AIx) which is an indicator of arterial stiffness and tone. The USCOM BP + has been validated for use in adults, pregnant women, and children [16–18]. Recruited participants were excluded if the signal-to-noise ratio, a quality control measure of cuff reading quality was < 6 [17]. Women provided fasting blood samples to assess blood glucose, insulin, lipids and C-reactive protein. Insulin resistance was calculated using the Homeostatic Model Assessment for Insulin Resistance (HOMA-IR) using fasting blood glucose and fasting insulin values [19]. Metabolic syndrome status at 3 years postpartum was defined based on the International Diabetes Federation (IDF) definition [20], which requires presence of central adiposity (defined by waist circumference which are ethnicity specific (for women of all ethnicities, this is ≥ 80 cm) and/or an obese BMI ≥ 30 kg/m^2^) and at least two of the following:

- Raised systolic blood pressure ≥ 130 mmHg or diastolic blood pressure ≥ 80 mmHg or treatment of previously diagnosed hypertension
- Raised serum triglycerides ≥ 1.7 mmol/L or being on medication for increased triglycerides
- Raised fasting plasma glucose ≥ 5.6 mmol/L or previously diagnosed type 2 diabetes mellitus
- Reduced HDL cholesterol ≤ 1.29 mmol/L

## Statistical analysis

Data were analysed using IBM SPSS Version 26. Women who were diagnosed with GDM in their index pregnancy were compared to those who were not (normoglycemic). Similarly, children who were born to mothers with GDM were compared for CVD risk factors with children who were born to mothers without GDM. Univariate analysis was used to compare anthropometric and baseline variables between GDM and normoglycemic pregnancies, with data presented as mean (SD) or *n* (%). Child variables were adjusted for child age, with the exception of BMI SDS as this has been adjusted for child age and sex already. As obesity is a significant predictor of both GDM and CVD [21, 22], secondary subgroup analysis was undertaken and both GDM and normoglycemic groups were stratified by obesity in early pregnancy (i.e. BMI ≥ 30 kg/m^2^) or non-obese (i.e. BMI ≤ 29.9 kg/m^2^). As the normoglycemic group includes women with other pregnancy complications that influence cardiovascular and metabolic health, to rule out any effect of these complications on the outcomes, exploratory analyses of cardiometabolic outcomes in pregnancy and 3 years postpartum were also performed in women with uncomplicated index pregnancies and their offspring.

For hemodynamic measures, blood pressure was measured in pregnant women who attended the study as per protocol. A proportion of women (*n* = 22, 7.8%) were pregnant at the time of follow-up and these women were excluded from the descriptive analysis of hemodynamic outcomes at 3 years postpartum. Linear regression analysis was undertaken to assess the association between developing GDM in the index pregnancy, and exposure to GDM in utero*,* and hemodynamic measurements compared to those with an uncomplicated pregnancy, with data presented as mean difference (95% CI). Adjustment was made for SEI and BMI in early pregnancy as both of these parameters influence both GDM and CVD development.

## Results

**Fig. 1:**
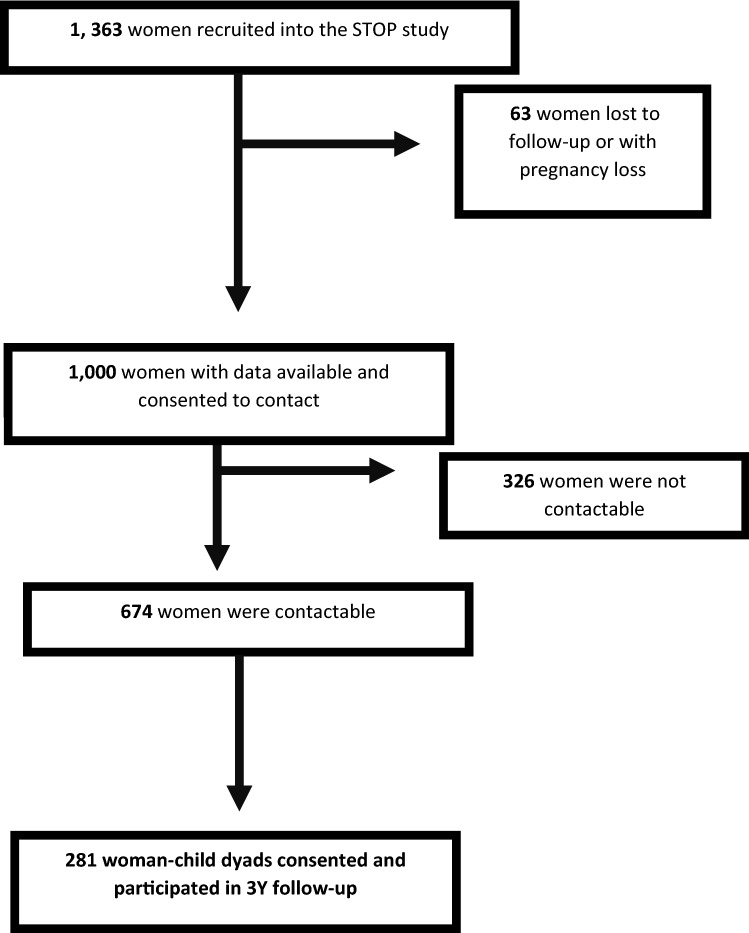
Flow chart of STOP follow-up participants

There were 1,363 women who participated in the STOP study. Figure 1 demonstrates the flow chart of participation in the follow-up study. There were 281 woman-child dyads who consented and participated in the follow-up study from January 2019 until June 2021. In the index pregnancy, 241 participants had a normoglycemic pregnancy and 40 participants experienced GDM. The participants who did not experience GDM (i.e. had a normoglycemic pregnancy) were comprised of women who had an uncomplicated pregnancy, or evidence of a maternal placental syndrome manifest as hypertensive disorder of pregnancy (i.e. preeclampsia or gestational hypertension), delivered preterm (< 37 weeks’ gestation) and/or delivered a small-for-gestational-age infant (below 10th customised percentile).

Those who attended the follow-up had a slightly higher BMI (28 ± 7.2 versus 27.9 ± 7.1 *p* = 0.02) than those who did not and although it is statistically significant, a 0.1 difference between in means is relatively minor. More women in the follow-up cohort were Caucasian (*n* = 246 (88.8%) versus *n* = 888 (81%) *p* < 0.0001 and more were university educated (*n* = 51 (8.4%) versus *n* = 154 (14.1%)) than non-attendees (Supplementary Table 1). However, they were not considered as confounding factors in the analysis due to the lack of evidence through the literature and the lack of association seen in Table 1 between those with a history of GDM and those without.

**Table 1:** Participant Demographics for women who participated in the STOP study and STOP 3Y Follow-Up Study

Characteristic*	GDM (*n* = 40)	Non-GDM (*n* = 241)	*p*-value
*Index pregnancy*			
Mean BMI (kg/m^2^)	30.8 (8.2)	27.4 (6.8)	**0.013**
Gravidity	1.85 (0.8)	2.05 (1.0)	0.924
SEI**	37.1 (16.8)	33.3 (13.6)	**0.016**
Caucasian ethnicity (n =)	35 (87.5%)	217 (89.3%)	0.731
Education status (*n* =)			**0.001**
Did not complete year 10	2 (5%)	3 (1.2%)	
Year 10	2 (5%)	17 (7%)	
Year 12	9 (22.5%)	31 (12.8%)	
Certificate	15 (37.5%)	92 (37.9%)	
Bachelor	10 (25%)	41 (16.9%)	
Higher degree	2 (5%)	7 (10%)	
*Pregnancy complication^*			
Uncomplicated	0	151 (62.1%)	**0.000**
Gestational hypertension	5 (12.5%)	13 (5.3%)	0.086
Preeclampsia	4 (10%)	25 (10.3%)	0.956
Preterm Birth	4 (10%)	10 (4.1%)	0.112
Small for gestational age	8 (20%)	29 (11.9%)	0.161
Child gestational age (weeks)	38.6 (2.1)	39.5 (1.7)	0.621
Child birthweight (g)	3202.8 (600)	3364.6 (501)	0.221
*3 years postpartum*			
Maternal age (years)	33 (5.6)	31 (4.9)	0.164
BMI (kg/m^2^)	29.7 (7.4)	29.1 (8.5)	0.891
Waist circumference(cm)	95 (21.1)	90 (19.4)	0.463

*Data are presented as Mean (SD) or *n* = (%)

**SEI is the New Zealand Socioeconomic Index on a scale of 10–90 with the lowest score indicating the person lives with the greatest disadvantage

^pregnancy complications are not mutually exclusive and participants may have experienced more than one pregnancy complication in index pregnancy

Those who attended the follow-up study who had GDM in their index pregnancy had significantly higher SEI than those with GDM who did not attend (37.1 ± 16.8 versus 33.4 ± 12.5 *p* = 0.001*,* on a scale of 10–90) (Supplementary Table 2).

Demographics of the participants who attended the 3-year follow-up are presented in Table 1. Those who had developed GDM had significantly higher SEI than those who did not have GDM in the index pregnancy (37.1 ± 16.8 versus 33.3 ± 13.6 *p* = 0.016). More women with a history of GDM had a bachelor’s degree than those without GDM (*p* = 0.001). BMI in early pregnancy was significantly higher in the GDM participants than normoglycemic participants (30.8 ± 8.2 versus 27.4 ± 6.8 *p* = 0.013) (Table 1).

### Cardiovascular risk factors during pregnancy and at 3 years postpartum

#### Baseline (9–16 weeks’ gestation)

Women with GDM during their pregnancy had higher mean systolic and diastolic blood pressure, mean arterial pressure, central systolic and central diastolic blood pressure at 9–16 weeks’ gestation compared to those who did not develop GDM in the index pregnancy (Table 2) (Supplementary Fig. 1). Exploratory analysis of GDM vs. uncomplicated pregnancy showed that mean systolic and diastolic blood pressure, mean arterial pressure, central systolic and central diastolic blood pressure were higher in those with GDM in index pregnancy compared to those with uncomplicated pregnancies (Table 2). As per protocol, fasting glucose at 28 weeks’ gestation was significantly higher in women with GDM compared to those with a normoglycemic pregnancy and those with an uncomplicated pregnancy (Table 2). Metabolic syndrome was more common in women with an uncomplicated pregnancy in early pregnancy than those who developed GDM (Table 2).

**Table 2:** Cardiovascular risk factors in women at baseline (9–16 weeks’), 34 weeks’ gestation and at 3 years postpartum

Baseline visit (9–16 weeks’ gestation)					
Variable	GDM (*n* = 40)	Normoglycemic pregnancy (*n* = 241)	*p*-value	Uncomplicated pregnancy (n = 149)	
Peripheral systolic blood pressure (mmHg)	120.9 (14.8)	114.6 (12.2)	0.056	112.3 (11.3)	**0.013**
Peripheral diastolic blood pressure (mmHg)	72.4 (10.9)	67.7 (8.2)	**0.012**	66.3 (7.7)	**0.004**
Mean arterial pressure (mmHg)	85.9 (12.1)	80.7 (9.0)	**0.002**	79 (8.2)	**0.000**
Augmentation Index (%)	36.5 (20.2)	32.0 (14.5)	0.125	47.6 (18.1)	0.160
Central systolic blood pressure (mmHg)	111.2 (13.7)	105.5 (11.2)	0.051	103.9 (11)	**0.036**
Central diastolic blood pressure (mmHg)	76.4 (9.7)	70.7 (7.6)	**0.030**	69.1 (7.8)	**0.009**

	GDM (*n* = 38)	Normoglycemic pregnancy (*n* = 219)	*p*-value	Uncomplicated pregnancy (*n* = 142)	*p*-value
Total cholesterol (mmol/L)	4.5 (0.7)	4.6 (0.7)	0.864	4.6 (0.7)	0.811
Triglycerides(mmol/L)	1.3 (0.5)	1.2 (0.4)	0.282	1.2 (0.5)	0.778
HDL-C(mmol/L)	1.6 (0.3)	1.6 (0.3)	0.890	1.6 (0.3)	0.784
CRP (mg/L)	4.8 (4.1)	5.2 (8.3)	0.383	4.3 (4.4)	0.895
Metabolic Syndrome (n (%))	13 (34.2%)	48 (21.9%)	0.084	24 (16.9%)	**0.024**

Third trimester (34 weeks’ gestation)					
	GDM (*n* = 18)	Normoglycemic pregnancy (*n* = 130)	*p*-value	Uncomplicated pregnancy (*n* = 77)	*p*-value
Peripheral systolic blood pressure (mmHg)	125.9 (11.8)	117.8 (11.1)	0.519	114.3 (9.5)	0.117
Peripheral diastolic blood pressure (mmHg)	76.4 (9.7)	70.7 (7.6)	0.251	68.7 (6.1)	**0.030**
Mean arterial pressure (mmHg)	90.9 (10.1)	83.3 (8.3)	0.271	80.9 (6.7)	**0.032**
Augmentation Index (%)	49.6 (15.4)	48.0 (17.7)	0.065	30.5 (15.4)	0.091
Central systolic blood pressure (mmHg)	113.8 (13)	106.0 (10.2)	0.168	102.9 (8.9)	**0.028**
Central diastolic blood pressure (mmHg)	79.6 (9.8)	73.9 (7.8)	0.260	71.9 (6.4)	**0.045**

3 years postpartum					
	GDM (*n* = 34)	Normoglycemic pregnancy (*n* = 202)	*p*-value	Uncomplicated pregnancy (*n* = 138)	*p*-value
Peripheral systolic blood pressure (mmHg)	121.2 (15.3)	120.6 (13.2)	0.270	119.4 (13.8)	0.487
Peripheral diastolic blood pressure (mmHg)	70.6 (12.3)	67.3 (11.2)	0.428	66.8 (12.3)	0.947
Mean arterial pressure (mmHg)	85 (14.4)	82.5 (11.7)	0.078	81.6 (12.3)	0.250
Augmentation Index (%)	52.5 (15.1)	55.3 (23.1)	0.076	53.5 (24.1)	0.078
Central systolic blood pressure (mmHg)	110.2 (16.6)	110.6 (12.4)	**0.046**	109.5 (13.2)	0.174
Central diastolic blood pressure (mmHg)	73.3 (12.7)	70.7 (10.6)	0.231	70 (11.5)	0.714

	GDM (*n* = 16)	Normoglycemic pregnancy (*n* = 66)	*p*-value	Uncomplicated (*n* = 44)	*p*-value
Fasting glucose(mmol/L)	4.8 (0.4)	4.6 (0.4)	0.995	4.4 (0.9)	0.686
Insulin (mU/L)	13.2 (9.5)	9.4 (6.1)	0.660	8.6 (5.0)	**0.022**
HOMA-IR	2.80 (2.2)	1.97 (1.3)	0.065	2.7 (6.4)	0.692
Triglycerides(mmol/L)	1.2 (0.6)	1.1 (0.6)	0.851	0.89 (0.4)	0.055
HDL-C(mmol/L)	1.4 (0.4)	1.4 (0.4)	0.638	2.6 (0.5)	0.722
LDL-C(mmol/L)	2.7 (0.5)	2.7 (0.7)	0.141	2.6 (0.5)	0.085
Total Cholesterol/HDL ratio	3.6 (1.0)	4.0 (3.9)	0.400	3.2 (0.7)	0.115
Non-HDL Cholesterol	3.3 (0.5)	3.1 (0.8)	0.067	3.1 (0.9)	0.686
Total Cholesterol(mmol/L)	4.7 (0.6)	5.2 (0.5)	0.367	4.5 (0.9)	0.174
CRP (mg/L)	4.02 (3.6)	6.52 (19.1)	0.311	6.7 (20.9)	0.323

Assessment of metabolic syndrome components in women at 3 years postpartum					
	GDM (*n* = 40)	Normoglycemic pregnancy (*n* = 237)	*p*-value	Uncomplicated (*n* = 146)	*p*-value
Abdominal obesity**	25 (62.5%)	128 (54%)	0.270	75 (53.1%)	0.738
Hypertension***	13 (32.5%)	53 (22.3%)	0.147	32 (21.9%)	0.137
Dysglycaemia^#^	1 (2.5%)	2 (0.8%)	0.341	0	0.159
Triglycerides > = 1.7 mmol/L	3 (7.5%)	6 (2.5%)	0.096	4 (2.7%)	0.721
Reduced HDL < 1.29 mmol/L	6 (15%)	28 (11.8%)	0.487	16 (10.9%)	0.759
Metabolic syndrome (n (%))	26 (65%)	130 (54.8%)	0.192	3 (2%)	**0.000**

*Results are reported as mean (SD)unless stated otherwise

**Abdominal obesity was waist circumference >  = 80 cm and/or obese BMI >  = 30 kg/m^2^

***Hypertension was defined as raised systolic blood pressure >  = 130 mmHg or diastolic blood pressure >  = 80 mmHg or treatment of previously diagnosed hypertension

^#^Dysglycaemia was defined as raised fasting plasma glucose >  = 5.6 mmol/L or previously diagnosed type 2 diabetes mellitus

Normoglycemic pregnancy includes those with other pregnancy complications including preeclampsia, gestational hypertension, spontaneous preterm birth and small for gestational age

#### 34 weeks’ gestation

Compared to women with uncomplicated pregnancies, women with GDM in their index pregnancy had significantly higher diastolic blood pressure, mean arterial pressure, central systolic and central diastolic blood pressure (Table 2).

#### 3 years postpartum

Central systolic blood pressure was higher in women with a history of GDM than in those with a normoglycemic pregnancy. Insulin was significantly higher in those with a history of GDM in pregnancy than those with an uncomplicated pregnancy (Table 2). The percentage with metabolic syndrome was significantly higher in women with a history of GDM compared to those with an uncomplicated index pregnancy. Only one participant who had hypertension at the time of the follow-up was taking antihypertensive medication. A history of GDM was associated with a 0.3 mmol/L increase in serum triglycerides at 3 years postpartum compared to history of uncomplicated pregnancy, after adjustment for covariates (Table 3).

**Table 3:** Association between GDM in pregnancy compared to uncomplicated pregnancy and subsequent cardiometabolic risk factors in mothers and children at 3 years post-pregnancy assessed by Linear regression

Outcomes	Adjusted Mean Difference (95% CI)*
Child waist circumference at 3 years**	1.9 (0.41 to 3.3)
Maternal Serum triglycerides at 3 years postpartum	**0.3 (0.07 to 0.6)**
Maternal Serum insulin at 3 years postpartum	1.9 (−1.5 to 5.2)

*Adjusted for maternal BMI and SEI in early pregnancy

**Also adjusted for child age

**Bold** indicates statistical significance

### Cardiovascular risk factors in children aged 3 years

Waist circumference was significantly greater in children exposed to GDM in utero compared to those who were born to mothers with a normoglycemic pregnancy and those born to mothers with an uncomplicated pregnancy (Table 4). However, this was attenuated by maternal BMI and SEI at early pregnancy.

**Table 4:** Cardiometabolic differences between children born to mothers with GDM compared to those who were not at 3 years postpartum

3-year follow-up					
	Children born to mothers with GDM (*n* = 33)	Children born to mothers with normoglycemic pregnancy (*n* = 198)	*p*-value*	Children born to mothers with uncomplicated pregnancies (*n* = 144)	*p*-value*
BMI SDS^	67 (28.7)	56.5 (30.7)	0.192	50.8 (32.6)	0.097
Waist circumference (cm)	53.6 (5)	51 (3.7)	**0.001**	51.2 (3.5)	**0.02**

	(*n* = 18)	(*n* = 107)		(*n* = 94)	
Systolic blood pressure (mmHg)	96.3 (18.6)	99.4 (14.0)	0.649	101.2 (13.1)	0.521
Diastolic blood pressure (mmHg)	56.1 (10.9)	57.7 (12)	0.905	57.0 (12.4)	0.826
Mean arterial pressure (mmHg)	69.0 (14.1)	71.3 (14.9)	0.842	72 (15.2)	0.889
Augmentation Index (AIx) (%)	89.6 (56.9)	82.5 (30.7)	0.979	89.1 (45)	0.914
Central systolic blood pressure (mmHg)	89.6 (15.3)	92.5 (15.2)	0.521	95.1 (15.6)	0.329
Central diastolic blood pressure (mmHg)	61.3 (10.4)	60.8 (11.1)	0.430	61.2 (12.0)	0.318

Reduced numbers for hemodynamic assessment due to non-compliance

Results are mean (SD) unless reported otherwise

*All outcomes except BMI SDS are corrected for child age

^BMI SDS is adjusted for child age and sex

### Effect of obesity in early pregnancy on CVD risk factors in women and children:

#### 9–16 weeks’ gestation

Amongst those who had a normoglycemic pregnancy, obese women had higher systolic blood pressure and mean arterial pressure than those who were not obese (Supplementary Table 3).

#### 34 weeks’ gestation

Augmentation Index was significantly higher in the obese women in the GDM group than in non-obese women with GDM. For those with a normoglycemic pregnancy, women who were obese had significantly higher systolic blood pressure, diastolic blood pressure, and mean arterial pressure, than those who were non-obese (Supplementary Table 3).

#### 3 years postpartum

Augmentation index in women with uncomplicated pregnancies was higher in those who were obese in early pregnancy compared to those who were not obese at the same timepoint. Those who were obese in the GDM group had significantly higher serum insulin, insulin resistance (HOMA-IR), LDL-C, and CRP, than those who were not obese. For those with a normoglycemic pregnancy, women who were obese in early pregnancy had significantly higher serum insulin, insulin resistance, total cholesterol/HDL ratio, and CRP than those who were not obese in early pregnancy. For women with an uncomplicated index pregnancy, those who were obese had significantly higher serum insulin, insulin resistance, and CRP levels than women who were not obese in early pregnancy (Supplementary Table 3).

#### Children aged 3

Children born to obese mothers with a normoglycemic pregnancy had higher diastolic blood pressure than those who were born to non-obese mothers. Children born to obese mothers with a normoglycemic pregnancy had a significantly higher waist circumference than children born to non-obese mothers with a normoglycemic pregnancy. Children born to obese mothers with an uncomplicated pregnancy had significantly higher BMI-SDS and waist circumference than those children born to non-obese mothers with an uncomplicated pregnancy.

## Discussion

Our observational follow-up study showed that serum triglycerides and insulin is higher triglycerides at 3 years postpartum compared to those with no history of GDM. However, the association between GDM and insulin was attenuated by maternal BMI and SEI in early pregnancy. Children exposed to GDM in utero had significantly higher waist circumference than children born to women with uncomplicated pregnancies but this was attenuated for the same covariates. Metabolic syndrome was higher in women with a history of GDM than those with uncomplicated pregnancy.

Obesity promotes development of insulin resistance and increases free fatty acids and inflammatory markers [23, 24]. There is discrepancy between studies regarding whether serum insulin levels are higher in women with previous GDM [25, 26] or if it is similar to controls [27–29]. Our subgroup analysis showed that obese women in each group (i.e. GDM, normoglycemic and uncomplicated index pregnancy) had elevated CRP, an inflammatory marker and insulin resistance. Therefore, obesity, together with history of GDM, may actually worsen metabolic health at an earlier time postpartum.

Serum triglyceride elevation at 3 years postpartum in women with a history of GDM supports that found in other studies [30]. Elevated serum triglycerides can be apparent 10 years before diagnosis of T2DM [31] and therefore may identify women who will develop T2DM later as glucose intolerance is associated with altered uptake of fatty acids.

A recent report showed that maternal glucose levels and BMI during pregnancy were independently associated with BMI, body fat and waist circumference in their exposed children at 11 years of age. However, combined exposure in utero increased the risk of obesity in the offspring further [32]. If there is an effect of GDM on childhood adiposity at 3 years of age, it is likely that our study was underpowered to assess this and further studies are required to look at this association.

This observational follow-up study has some strengths. Women in the original STOP study included only nulliparous women and excluded women with serious medical conditions or at high risk of pregnancy complications due to underlying conditions, allowing us to assess the effect of pregnancy complications without confounding by greater parity in a cohort of young women. They are generally overlooked in cardiovascular risk assessment as heart attack statistical models are targeted to older age groups. We assessed haemodynamic and metabolic risk factors non-invasively in women and haemodynamics in their children at 3 years of age. These non-conventional vascular assessments have seldom been reported in the literature for women, and particularly in early childhood.

Our study assessed women and children from a hospital servicing a disadvantaged population and highlights impact of socioeconomic disadvantage on cardiovascular risk factors in young women and their children. The high incidence of obesity in early pregnancy in participants in the STOP Study makes it possible that many women may have entered pregnancy with undiagnosed insulin resistance and glucose intolerance making a diagnosis of GDM more likely. We recommend future larger studies in women and young children in disadvantaged communities to confirm or refute our findings.

Our study has some limitations. Approximately one quarter of participants from the original STOP study attended the 3-year follow-up. Majority of this loss is due to loss of contact. Indeed 42% of the women who were contactable agreed to participate. The difficulties associated with living with disadvantage, reduce the likelihood that such a population will participate in clinical research [33]. Therefore, there may be risk of selection bias in our study. Although we have shown statistically significant differences in some parameters these are relatively small. This may simply reflect the fact that 3 years postpartum may be very early in the progression to CVD. Nevertheless, these small metabolic changes may amplify over time.

The observational nature of the study means that we cannot infer causality. Only 82 women completed a fasting blood test, and some data are missing for anthropometric and hemodynamic measures in the offspring due to non-compliance. Missing data for fasting serum parameters may mean that the rate of metabolic syndrome in the cohort could be underreported. Some women who attended the follow-up were pregnant (*n* = 22, 7.8%) and therefore 3 years postpartum data were missing for these participants. We recommend further longitudinal assessments in a larger, better powered cohort to determine whether cardio-metabolic changes exacerbate in the long term.

## Conclusion

Cardiovascular risk factors in women with a history of GDM and their offspring are present at 3 years after delivery, with maternal BMI and SEI in early pregnancy either mediating or attenuating these associations. Our data warrant larger, more highly powered and longitudinal studies of cardiometabolic health in women and children exposed to GDM. Our study suggests that early interventions for socioeconomically disadvantaged young women and children may be important to improving long term health in communities that are known to have high rates of chronic diseases.

## Supplementary Information

Below is the link to the electronic supplementary material.

Supplementary file1 (DOCX 49 KB)
