# Supplementary material for: Gestational diabetes mellitus and cardio-metabolic risk factors in women and children at 3 years postpartum
Source: Acta Diabetol. 2022 Jul 11;59(9):1237–46. doi: 10.1007/s00592-022-01914-y (PMC9329416; doi:10.1007/s00592-022-01914-y)
Supplement: Supplementary file 1 — Supplementary file1 (DOCX 49 KB) [file 592_2022_1914_MOESM1_ESM.docx]

**Supplementary Table 1:** Demographics of participants who did attend and did not attend 3 year follow-up

| **Characteristic*** | **Follow-Up (n=277)** | **Not followed up (1017)** | **p-value** |
| --- | --- | --- | --- |
| **Index pregnancy** | | | |
| Age at recruitment | 25.7 (5) | 27.1 (5.1) | 0.807 |
| BMI | 28 (7.2) | 27.9 (7.1) | **0.020** |
| SEI | 33.8 (14.2) | 32.7 (13.9) | 0.172 |
| Caucasian ethnicity | 246 (88.8%) | 888 (81%) | **0.000** |
| Education Status  Did not complete year 10 Year 10 Year 12  Certificate  Bachelor  Higher degree | 5 (1.8%) 32 (11.6%) 32 (11.6%) 51 (36.8%) 51 (18.4%) 19 (6.9%) | 24 (2.2%) 229 (20.9%) 229 (20.9%) 286 (35.2%) 154 (14.1%) 61 (5.6%) | **0.000** |
| Pregnancy complication^ GDM Gestational hypertension Preeclampsia Preterm Birth Small for gestational age | 39 (14.1%) 18 (6.5%) 28 (10.1%) 14 (5.1%) 37 (13.4%) | 160 (15.7%) 70 (6.9%) 93 (9.1%) 48 (4.7%) 116 (11.4%) | 0.285 0.822 0.622 0.815 0.376 |
| Child gestational age (weeks) | 39.4 (1.8) | 39.2 (2.0) | 0.944 |
| Child birthweight (g) | 3336.17 (520.2) | 3347.3 (563.2) | 0.420 |

Results are reported as mean (SD) or n= (%)
^ pregnancy complications are not mutually exclusive therefore one participant may experience multiple pregnancy complications

Supplementary Table 2: Differences in attendees and non-attendees for STOP 3 year follow-up

| All participants | | |
| --- | --- | --- |
|  | Mean (SD) | P value |
| NZSEI | Attended: 33.8 (14.0) Did not attend: 32.7 (13.9) | 0.172 |
| Age (years) | Attended: 31.2 (5.0) Non attendee: 29.6 (6.7) | 0.387 |
| Booking BMI (kg/m2) | Attended: 27.8 (7.2) Did not attend:: 28 (7.1) | 0.648 |
| GDM participants only | | |
| NZSEI | Attended: 36.9 17.3 Non attendee: 33.4 12.5 | 0.002 |
| Booking BMI (kg/m2) | Attended 31 8.3 Did not attend: 32.1 8.8 | 0.697 |

**Supplementary Table 3:** Subgroup analysis of obesity at index pregnancy on cardiovascular risk factors in women and children

| **Baseline visit (9-16 weeks’ gestation)** | | | | | | | | | | | | | |
| --- | --- | --- | --- | --- | --- | --- | --- | --- | --- | --- | --- | --- | --- |
| **Variable** | **GDM (n=40)** | | | | **Normoglycemic pregnancy (n=241)** | | | | **Uncomplicated (n=151 )** | | | | |
|  | **Obese**  **(n=21)** | | **Non-obese (n=19)** | **p-value** | **Obese (n=62)** | **Non-obese (n=179)** | | **p-value** | **Obese (n=27)** | | **Non-obese (n=124)** | | **p-value** |
| Peripheral systolic blood pressure (mmHg) | 127.5 (13.8) | 113.6 (12.6) | | 0.173 | 122.4 (12.8) | 111.8 (10.8) | | **0.034** | 119.0 (11.7) | | 110.9 (10.6) | | 0.425 |
| Peripheral diastolic blood pressure (mmHg) | 77.5 (11.1) | 66.9 (7.7) | | 0.124 | 72.5 (9.3) | 66.1 (7.2) | | 0.052 | 71 (8.5) | | 65.3 (7.1) | | 0.512 |
| Mean arterial pressure (mmHg) | 91.6 (11.8) | 79.5 (9.0) | | 0.214 | 86.6 (10.2) | 78.6 (7.7) | | **0.014** | 85 (8.3) | | 77.7 (7.6) | | 0.463 |
| Augmentation Index (%) | 50.1 (16.7) | 49.2 (14.3) | | 0.344 | 43.8 (16.1) | 49.5 (18) | | 0.101 | 43.2 (18.1) | | 48.6 (17.9) | | 0.584 |
| Central systolic blood pressure (mmHg) | 117.2 (12.7) | 104.5 (11.6) | | 0.610 | 111.6 (11.4) | 103.4 (10.4) | | 0.161 | 109 (10.9) | | 102.2 (10.7) | | 0.696 |
| Central diastolic blood pressure (mmHg) | 80.4 (10.9) | 69.8 (7.6) | | 0.148 | 75.7 (9.4) | 69 (7.4) | | 0.106 | 73.4 (8.5) | | 67.8 (7.2) | | 0.557 |
|  | **Obese (n=20)** | **Non-obese (n=17)** | | **p-value** | **Obese (n=25)** | **Non-Obese (n=117)** | | **p-value** | **Obese (n=25)** | | **Non-obese (n=117)** | | **p-value** |
| Total cholesterol (mmol/L) | 4.7 (0.8) | 4.4 (0.6) | | 0.446 | 4.7 (0.5) | 4.6 (0.8) | | 0.055 | 4.7 (0.5) | | 4.6 (0.8) | | 0.055 |
| Triglycerides(mmol/L) | 1.4 (0.6) | 1.3 (0.4) | | 0.119 | 1.2 (0.4) | 1.2 (0.5) | | 0.509 | 1.2 (0.4) | | 1.2 (0.5) | | 0.509 |
| HDL-C(mmol/L) | 1.5 (0.3) | 1.7 (0.3) | | 0.671 | 1.5 (0.3) | 1.6 (0.3) | | 0.537 | 1.5 (0.3) | | 1.6 (0.3) | | 0.537 |
| CRP | 5.3 (4.8) | 4.1 (3.2) | | 0.007 | 6.1 (10) | 4.8 (7.6) | | 0.164 | 4.9 (6.1) | | 4.2 (3.9) | | 0.239 |
| **Third trimester (34 weeks’ gestation)** | | | | | | | | | | | | | |
|  | **GDM (n=18)** | | | | **Normoglycemic pregnancy (n=130)** | | | | **Uncomplicated (n=77)** | | | | |
|  | **Obese**  **(n=11)** | **Non-obese (n=7)** | | **p-value** | **Obese (n=35)** | **Non obese (n=95)** | | **p-value** | **Obese (n=14)** | | **Non-obese (n=63)** | | **p-value** |
| Peripheral systolic blood pressure (mmHg) | 129.8 (9.6) | 119.9 (13) | | 0.699 | 126.4 (11.1) | 114.6 (9.3) | | **0.042** | 122.3 (9.3) | | 112.6 (8.7) | | 0.595 |
| Peripheral diastolic blood pressure (mmHg) | 77.6 (7.8) | 74.6 (12.6) | | 0.350 | 74.5 (9.3) | 69.3 (6.4) | | **0.004** | 71.6 (6.5) | | 68.1 (5.9) | | 0.483 |
| Mean arterial pressure (mmHg) | 92.4 (8.2) | 88.7 (13) | | 0.589 | 88.5 (9.8) | 81.4 (6.9) | | **0.012** | 84.9 (7.5) | | 80 (6.3) | | 0.322 |
| Augmentation Index (%) | 34.9 (14.2) | 39 (28.5) | | **0.049** | 33.9 (16.1) | 31.3 (14) | | 0.674 | 28.4 (14.5) | | (31 (14.7) | | 0.844 |
| Central systolic blood pressure (mmHg) | 117.3 (10) | 108.6 (15.9) | | 0.673 | 113.6 (10.3) | 103.3 (8.8) | | 0.065 | 109.9 (8.7) | | 101.4 (8.2) | | 0.841 |
| Central diastolic blood pressure (mmHg) | 81.0 (8.1) | 77.4 (12.3) | | 0.483 | 78.1 (9.3) | 72.3 (6.6) | | **0.006** | 75.2 (6.8) | | 71.1 (6.2) | | 0.496 |
| **3 years postpartum (women)** | | | | | | | | | | | | | |
|  | **GDM (n=38)** | | | | **Normoglycemic pregnancy (n=202)** | | | | **Uncomplicated (n=137)** | | | | |
|  | **Obese (n=20)** | **Non-obese (n=14)** | | **p-value** | **Obese (n=53)** | **Non-obese (n=149)** | | **p-value** | **Obese (n=26)** | | **Non-obese (n=111)** | | **p-value** |
| Peripheral systolic blood pressure (mmHg) | 127.0 (15.7) | 113 (10.6) | | 0.251 | 125.9 (14.8) | 118.7 (12.2) | | 0.268 | 125.8 (16.3) | | 117.3 (12.1) | | 0.127 |
| Peripheral diastolic blood pressure (mmHg) | 74.5 (13.1) | 65.5 (8.9) | | 0.203 | 72 (12.1) | 66.2 (10.5) | | 0.217 | 71.5 (12.8) | | 65.3 (11.4) | | 0.562 |
| Mean arterial pressure (mmHg) | 91.2 (14.6) | 76.3 (8.9) | | 0.090 | 88 (12.8) | 80.5 (10.6) | | 0.135 | 88.6 (12.1) | | 79.5 (11.3) | | 0.691 |
| Augmentation Index (%) | 56.1 (13.1) | 47.2 (16.7) | | 0.511 | 58.9 (29.3) | 54.0 (20.5) | | 0.150 | 59.4 (35.7) | | 52.1 (20.5) | | **0.040** |
| Central systolic blood pressure (mmHg) | 115.7 (18.2) | 102.8 (10.7) | | 0.274 | 115.5 (13.5) | 108.9 (11.6) | | 0.356 | 115.1 (15.3) | | 107 (11.9) | | 0.280 |
| Central diastolic blood pressure (mmHg) | 77.3 (14) | 68.2 (8.8) | | 0.083 | 74.9 (12.1) | 69.2 (9.6) | | 0.093 | 74.8 (13.4) | | 68.4 (10.5) | | 0.182 |
|  | | | | | | | | | | | | |  |
|  | **GDM (n= 16)** | | | | **Normoglycemic pregnancy n= 69)** | | | | **Uncomplicated (n=41)** | | | | |
| Characteristic | **Obese (n=10)** | **Non-obese (n=6)** | | **p-value** | **Obese (n=24)** | **Non-Obese (n=45)** | | **p-value** | **Obese (n=9)** | | **Non-obese (n=32)** | | **p-value** |
| Insulin (mU/L) | 16.7 (10.9) | 7.9 (2.8) | | **0.022** | 13.5 (7.11) | 7.4 (3.9) | **0.004** | 14.3 (7.2) | | 7.1 ^34^ | | **0.000** | |
| HOMA-IR | 3.6 (2.5) | 1.7 (0.6) | | **0.032** | 4.7 (8.8) | 1.5 (0.9) | **0.009** | 7.4 (13.3) | | 1.4 (0.6) | | **0.000** | |
| Triglycerides(mmol/L) | 1.4 (0.4) | 1.3 (0.6) | | 0.385 | 1.4 (0.8) | 0.87 (0.4) | **0.000** | 1.2 (0.4) | | 0.8 (0.3) | | 0.815 | |
| HDL-C(mmol/L) | 1.3 (0.4) | 1.5 (0.4) | | 0.968 | 1.3 (0.8) | 1.4 (0.3) | 0.238 | 1.3 (0.3) | | 1.4 (0.3) | | 0.803 | |
| LDL-C(mmol/L) | 2.7 (0.6) | 2.7 (0.1) | | **0.046** | 2.9 (0.6) | 2.6 (0.7) | 0.476 | 3.1 (0.9) | | 2.5 (0.7) | | 0.640 | |
| Total Cholesterol/HDL ratio | 3.8 (1.1) | 3.3 (0.7) | | 0.437 | 5.1 (5.8) | 3.3 (0.8) | **0.015** | 3.6 (0.6) | | 3.1 (0.7) | | 0.631 | |
| Non-HDL Cholesterol | 3.4 (0.7) | 3.2 (0.2) | | 0.071 | 3.6 (0.9) | 3.0 (0.8) | 0.676 | 3.8 (1) | | 2.9 (0.8) | | 0.329 | |
| Total Cholesterol(mmol/L) | 4.7 (0.6) | 4.8 (0.4) | | 0.396 | 5.1 (5.8) | 3.2 (0.9) | 0.634 | 5.0 (1.2) | | 4.3 (0.8) | | 0.389 | |
| CRP (mmol/L) | 5.4 (4) | 1.9 (1.2) | | **0.048** | 11.7 (29.8) | 3.4 (5.5) | **0.026** | 19.5 (43) | | 2.5 (2.6) | | **0.001** | |
| **3 years post pregnancy (children)** | | | | | | | | | | | | | |
|  | **Children born to mothers with GDM (n=33)** | | | | **Children born to mothers with normoglycemic pregnancy (n=220)** | | | **Children born to mothers with uncomplicated pregnancy (n=121)** | | | | | |
|  | **Obese (n=18)** | **Non-obese (n=18)** | | **p-value*** | **Obese (n=56)** | **Non-obese (n=164)** | **p-value*** | **Obese (n=22)** | | **Non-obese (n=99)** | | **p-value*** | |
| BMI SDS ^ | 70.5 (32.4) | 58.3 (27.4) | | 0.221 | 64.7 (30) | 50.8 (31.3) | 0.209 | 65.2 (32.3) | | 48 (32.1) | | **0.005** | |
| Waist circumference | 55.4 (5.4) | 52.1 (4.1) | | 0.079 | 52.5 (3.8) | 50.6 (3.7) | **0.01** | 53.4 (3.7) | | 50.7 (3.3) | | **0.001** | |
|  | **(n=8)** | **(n=14)** | |  | **(n=43)** | **(n=113)** |  | **(n=16)** | | **(n=73)** | |  | |
| Systolic blood pressure (mmHg) | 100.3 hi(14.3) | 98.2 (18.3) | | 0.929 | 101.6 (14.2) | 100.1 (14.6) | 0.666 | 102.50  (12.4) | | 100.7 (13.4) | | 0.579 | |
| Diastolic blood pressure (mmHg) | 59 (7.6) | 58 (14) | | 0.739 | 60.3 (13) | 57.1 (16.2) | 0.171 | 58.2 (10.5) | | 57.7 (12.8) | | 0.836 | |
| Mean arterial pressure (mmHg) | 79.3 (19.1) | 68.9 (15) | | 0.152 | 75.1 (15.6) | 70.4 (15) | 0.108 | 73.6 (12.8) | | 71.6 (15) | | 0.575 | |
| Augmentation Index (%) | 86.4 (33.6) | 89.7 (62.5) | | 0.934 | 84.4 (46.1) | 90.1 (39.7) | 0.540 | 91.9 (61.8) | | 59.4 (41.6) | | 0.756 | |
| Central systolic blood pressure (mmHg) | 91.2 (12.8) | 92.1 (15.2) | | 0.894 | 95.3 (15.2) | 93.3 (15.2) | 0.512 | 98.8 (22.5) | | 94.3 (13.8) | | 0.294 | |
| Central diastolic blood pressure (mmHg) | 65.8 (15.2) | 63.3 (13.3) | | 0.986 | 66.2 (14.5) | 60 (11.8) | **0.010** | 63.2 (13.4) | | 60.7 (11.8) | | 0.482 | |

Results are mean (SD) unless otherwise stated

**B**

**A**

**C**

**D**

**Figure 2.** Means of peripheral and central measures at 12 weeks’ gestation, 34 weeks’ gestation and 3 years postpartum for women with a history of gestational diabetes mellitus (GDM) and those without a history of gestational diabetes mellitus (nGDM); SBP – systolic blood pressure, DBP – diastolic blood pressure, cSBP – central systolic blood pressure, cDBP – central diastolic blood pressure
